# Thermal-stress-induced birefringence management of complex laser systems by means of polarimetry

**DOI:** 10.1038/s41598-022-22698-9

**Published:** 2022-10-31

**Authors:** Ondřej Slezák, Magdalena Sawicka-Chyla, Martin Divoký, Jan Pilař, Martin Smrž, Tomáš Mocek

**Affiliations:** HiLASE Centre, Institute of Physics, Czech Academy of Sciences, Za Radnicí 828, 252 41 Dolní Břežany, Czech Republic

**Keywords:** Applied optics, Lasers, LEDs and light sources, Optical techniques

## Abstract

The novel method of the thermally-induced polarization changes driven power losses (TIPCL) analysis in the complex laser systems has been developed. The measurement has been tested on the amplifier chain of the 100 J / 10 Hz laser system ‘Bivoj’ operated at HiLASE Centre. By the usage of the measured non-uniform Mueller matrix of the amplifier chain, the optimization of the ideal input and output polarization state has been calculated numerically. The results of the optimization have been applied to the laser system, thus reducing the TIPCL from originally observed more than 33% to 7.9% for CW beam and to 9% for pulsed laser beam, respectively. To the best of our knowledge, this result represents the most efficient TIPCL suppression method for complex laser systems so far. The method also allows the definition of the ideal fully polarized non-uniform pre-compensation of input beam consequently suffering from zero TIPCL.

## Introduction

Laser beams in high energy high average power lasers suffer from non-uniform polarization change during the amplification. This polarization change originates from stress induced birefringence caused by heat load occurring dominantly in the amplifier. Such polarization changes reduce energy available to polarization sensitive experiments such as harmonic conversion^1^ or optical parametric amplification^2–4^. Losses in such cases can be as high as 50%. Another deleterious effect of the thermally induced polarization change is the degradation of the beam profile when the beam passes through any diattenuator, typically linear polarizer. It has been shown recently, that the losses can be substantially mitigated by the optimization of input linear or circular polarization state^5^, by the proper design of the laser amplifiers and their cooling^6,7^, by the mutual compensation of thermally induced birefringence realized between multiple passes through the same heated optical elements^8–10^, by proper orientation of the input linear polarization in square-shaped amplifiers^11^, or by the suitable crystal orientation of the amplifiers^12^. Even so, the methods based on the results referenced above are usually not sufficient to keep the power losses at acceptable values allowing efficient laser system operation. This is due to the presence of many polarization-affecting optical components in the system, due to the differences in optical paths between the single passes through the amplifier head e.g. in geometrical multi-passes and other effects.

For further reduction of the TIPCL one would need to optimize the output polarization state as well as the input. Moreover, the general elliptical polarization states should be considered for complex amplifier chains. Such optimization process is, however, at least four-parameter optimization (ellipticity angle and azimuth of both, input and output polarization), which is experimentally demanding in the terms of time required for finding the optimal set of these parameters. Our proposal is the employment of spatially resolved Mueller matrix polarimetry applied to the whole laser system which allows the transfer of the time consuming four parameter optimization process from large number of experimental measurements into the numerical calculation. Such process can be then very fast and efficient in finding the optimal input polarization state and the output polarization state change leading to the maximal suppression of these non-uniform polarization state changes.

Both the measurement and the optimization process has been applied and tested experimentally on the 100 J/10 Hz laser chain ‘Bivoj’ which is located at HiLASE centre. The laser is operated at 1029.8 nm wavelength with 10 ns pulse duration. The output beam is square-shaped 75 × 75 mm^2^. It is the first kilowatt-class high energy laser^13,14^ that recently increased its output power to almost 1.5 kW^15^.

**Figure 1:**
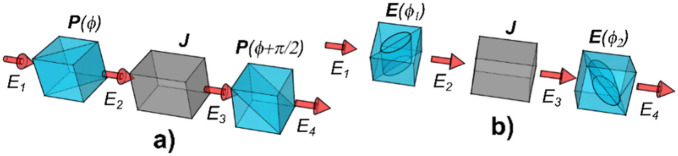
The schematics of the TIPCL evaluation. (**a**) Traditional definition as the throughput of the system ***J*** placed between crossed linear polarizers ***P***, (**b**) generalized definition as the throughput of the system ***J*** placed between two elliptical polarizers ***E***.

The development of the optimization process has given rise to the novel method of TIPCL reduction. It is based on the generalized definition of TIPCL which is, in terms of power loss, equivalent to the measurement of the power loss of the system placed between two arbitrarily oriented elliptical polarizers. On the contrary, the traditional conception defines the TIPCL (also called depolarization losses) as the throughput of crossed linear polarizers^16^. Both TIPCL definitions are schematically shown in the Fig. 1.

Considering that the polarization state after the distorting system ***J*** is described by non-uniform Jones vector ${E}_{3}={E}_{3}\left(x,y\right)$ shown in the Fig. 1, there will always be some nonzero transmission through the output polarizer. The Jones matrix of the ideal polarizer is an orthogonal projector and therefore it is Hermitian and idempotent. These two properties lead to the following evaluation: case a) gives the output linear polarization in the form ${E}_{4}={\varvec{P}}\left(\phi +\pi /2\right){E}_{3}$ and the output intensity ${I}_{a}={E}_{3}^{\dagger}{\varvec{P}}\left(\phi +\pi /2\right){E}_{3}$, where the cross sign stands for the conjugate transpose. The similar applies to the case b) containing elliptical polarizers ***E***, ${E}_{4}={\varvec{E}}\left({\phi }_{2}\right){E}_{3}$ and ${I}_{b}={E}_{3}^{\dagger}{\varvec{E}}\left({\phi }_{2}\right){E}_{3}$.

## Mueller matrix polarimetry

The Mueller matrix polarimetry is a method of measurement of a complete set of polarization properties of transmissive or reflective samples^17^. However, to the best of our knowledge, it has never been used for the analysis of the polarization properties of the whole laser system amplifier chain in order to study the thermo-optical effects. The measurement method is evaluating the response of the system to several different input polarization states and subsequently quantifies the polarization change obtained in the system by the reconstruction of the complete polarization transmission operator, e.g. Mueller matrix. The optical path through amplifier chain contains approximately 120 optical elements and it is difficult to evaluate their polarization properties separately. Nevertheless, the proposed measurement contains all the phenomena taking part in the formation of the resulting polarization state of the laser beam.

**Figure 2:**
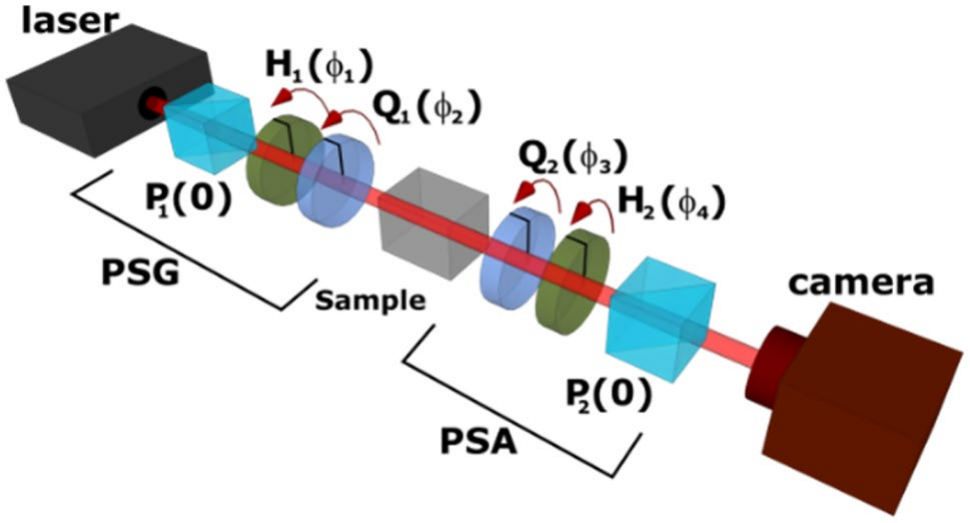
Mueller matrix polarimeter. *PSG* polarization state generator, *PSA* polarization state analyzer; P_1_, P_2_—polarizers; H_1_, H_2_—half-waveplates; Q_1_, Q_2_—quarter-waveplates. The angles φ denotes the inclination of the waveplates fast axes from the vertical direction.

The experimental setup used in this particular case for the Mueller matrix polarimetry of the laser system is shown in the Fig. 2.

An arbitrary polarization state can be generated by the polarization state generator PSG. The probe laser beam is polarized to the vertical linear polarization by the high-contrast polarizer P_1_ and the desired polarization state is then generated by the set of waveplates H_1_ and Q_1_. The polarized beam is subsequently propagated through the sample, where it accumulates the information about the polarization properties of the sample. The part of this information is then transformed into the laser intensity in the polarization state analyzer PSA, consisting of two waveplates Q_2_ and H_2_ and the vertically oriented high-contrast linear polarizer P_2_. It should be noted that the sample was in this particular case the whole laser system.

All four quartz true zero-order waveplates used in the polarimeter were placed in the motorized mounts which allow precise computer-controlled reorientation.

The experimental scheme shown in the Fig. 2 will be used as both, the Mueller-matrix polarimeter and TIPCL compensation scheme, respectively. For the case of compensation scheme the equivalency to the Fig. 1 b) in terms of the measured intensity should be shown. The Jones vector incident on the camera can be expressed as ${E}_{4}={{\varvec{P}}}_{2}{\varvec{U}}{E}_{3}$, where ${E}_{3}$ denotes the Jones vector of the wave leaving the sample and ***U*** stands for the unitary Jones matrix representing the combination of waveplates ***Q***_2_ and ***H***_2_^18^. The intensity recorded on the CCD (AVT Manta 125B) is in this case given by $I={E}_{3}^{\dagger}{{\varvec{U}}}^{\dagger}{{\varvec{P}}}_{2}{\varvec{U}}{E}_{3}$. The matrix ${{\varvec{U}}}^{\dagger}{{\varvec{P}}}_{2}{\varvec{U}}$ is similar to the linear polarizer matrix ***P***_2_, thus having the same spectrum, but the eigenstates are transformed by the ***U*** matrix from the linear ones to general elliptical ones. Therefore, the intensity *I* is equivalent to *I*_b_ derived for the two elliptical polarizers case, even if every scheme produces different output polarization.

The intensity profile which can be observed by the camera is given by:

1 $$I\left(x,y\right)={{I}_{0}\left(x,y\right)A}^{T}\left({\phi }_{3},{\phi }_{4}\right){\varvec{M}}\left(x,y\right)G\left({\phi }_{1},{\phi }_{2}\right),$$

where ***M****(x,y)* is a general spatially non-uniform Mueller matrix characterizing the amplifier chain polarization properties, *A* and *G* are the column vectors derived from the Mueller matrices of the PSA and PSG, respectively. Vector *A* is the first column of the Mueller matrix ***A*** describing the PSA

2a $${\varvec{A}}\left({\phi }_{3},{\phi }_{4}\right)={\varvec{P}}\left(0\right){\varvec{H}}\left({\phi }_{4}\right){\varvec{Q}}\left({\phi }_{3}\right),$$

2b $$A\left({\phi }_{3},{\phi }_{4}\right)=\frac{1}{2}\left[\begin{array}{c}\begin{array}{c}1\\ -\frac{1}{2}\left\{\mathrm{cos}4{\phi }_{4}+\mathrm{cos}\left[4\left({\phi }_{4}-{\phi }_{3}\right)\right]\right\}\end{array}\\ \begin{array}{c}-\frac{1}{2}\left\{\mathrm{sin}4{\phi }_{4}-\mathrm{sin}\left[4\left({\phi }_{4}-{\phi }_{3}\right)\right]\right\}\\ \mathrm{sin}\left[2\left(2{\phi }_{4}-{\phi }_{3}\right)\right]\end{array}\end{array}\right],$$

where ***P***, ***H***, and ***Q*** denotes the Mueller matrices of the polarizer, half-wave plate, and quarter-wave plate, respectively. The vector G characterizes the PSG in similar way including the input polarization state characterized by its Stokes vector *S*_*in*_

3a $$G\left({\phi }_{1},{\phi }_{2}\right)={\varvec{Q}}\left({\phi }_{2}\right){\varvec{H}}\left({\phi }_{1}\right){\varvec{P}}\left(0\right){S}_{in}={\varvec{G}}\left({\phi }_{1},{\phi }_{2}\right){S}_{in},$$

3b $$G\left({\phi }_{1},{\phi }_{2}\right)=\left[\begin{array}{c}\begin{array}{c}1\\ -\frac{1}{2}\left\{\mathrm{cos}4{\phi }_{1}+\mathrm{cos}\left[4\left({\phi }_{2}-{\phi }_{1}\right)\right]\right\}\end{array}\\ \begin{array}{c}-\frac{1}{2}\left\{\mathrm{sin}4{\phi }_{1}+\mathrm{sin}\left[4\left({\phi }_{2}-{\phi }_{1}\right)\right]\right\}\\ \mathrm{sin}\left[2\left({\phi }_{2}-{2\phi }_{1}\right)\right]\end{array}\end{array}\right].$$

It is needed to define the 16 sets of four waveplate angles. Two angles ${\phi }_{1}$, ${\phi }_{2}$ provides the values of *G* vector given by (3b), while the other couple ${\phi }_{3}$, ${\phi }_{4}$ defines the values of *A* given by (2b). Let us organize these values into the ***G*** and ***A*** 4 × 4 matrix (*A* or *G* vector for each ${\phi }_{3}$, ${\phi }_{4}$ or ${\phi }_{1}$, ${\phi }_{2}$ couple forms the columns of the corresponding matrix). The angles $\phi$ should be chosen so that the matrices ***G*** and ***A*** are both non-singular. The experimentally measured intensities then form the ***I*** matrix

4 $${\varvec{I}}\left(x,y\right)={{I}_{0}\left(x,y\right){\varvec{A}}}^{{\varvec{T}}}{\varvec{M}}\left(x,y\right){\varvec{G}}.$$

The 16 components of the ***I*** matrix are to be determined experimentally. Once the measured intensities are arranged into the ***I*** matrix, the Mueller matrix of the sample can be reconstructed as

5 $${I}_{0}\left(x,y\right){\varvec{M}}\left(x,y\right)={{\varvec{A}}}^{-{\varvec{T}}}{\varvec{I}}\left(x,y\right){{\varvec{G}}}^{-1}.$$

One of the possible choices of all 16 sets of waveplate angles is summarized in the Table 1.

**Table 1:** Angles of the polarimeter waveplates [rad].

	*I*_11_	*I*_12_	*I*_13_	*I*_14_	*I*_21_	*I*_22_	*I*_23_	*I*_24_	*I*_31_	*I*_32_	*I*_33_	*I*_34_	*I*_41_	*I*_42_	*I*_43_	*I*_44_
${\phi }_{1}$	0	0	0	0	0	0	0	0	π/8	π/8	π/8	π/8	π/4	π/4	π/4	π/4
${\phi }_{2}$	0	0	0	0	π/4	π/4	π/4	π/4	π/4	π/4	π/4	π/4	0	0	0	0
${\phi }_{3}$	0	0	π/4	π/2	0	0	π/4	π/2	0	0	π/4	π/2	0	0	π/4	π/2
${\phi }_{4}$	0	π/8	π/8	π/4	0	π/8	π/8	π/4	0	π/8	π/8	π/4	0	π/8	π/8	π/4

Such choice of waveplate angles leads, through the Eqs. (2b) and (3b), to the ***A*** and ***G*** matrices (6). The advantage of the choice of the $\phi$ angles summarized in Table 1 is now obvious, because for this particular choice

6 $${\varvec{A}}=\frac{1}{2}{\varvec{G}}=\frac{1}{2}\left[\begin{array}{cc}\begin{array}{cc}1& 1\\ -1& 0\end{array}& \begin{array}{cc}1& 1\\ 0& 1\end{array}\\ \begin{array}{cc}0& 0\\ 0& 1\end{array}& \begin{array}{cc}-1& 0\\ 0& 0\end{array}\end{array}\right]\boldsymbol{ }\Rightarrow \boldsymbol{ }{{\varvec{A}}}^{-1}=2{{\varvec{G}}}^{-1},$$

Therefore the intensity and the Mueller matrices of the sample are given by

7 $${\varvec{I}}=\frac{1}{2}{{\varvec{G}}}^{{\varvec{T}}}{\varvec{M}}{\varvec{G}}\boldsymbol{ }\Rightarrow \boldsymbol{ }{\varvec{M}}=2{{\varvec{G}}}^{-{\varvec{T}}}{\varvec{I}}{{\varvec{G}}}^{-1},$$

where

8 $${{\varvec{G}}}^{-1}=\frac{1}{2}\left[\begin{array}{cc}\begin{array}{cc}1& -1\\ 0& 0\end{array}& \begin{array}{cc}1& -1\\ 0& 2\end{array}\\ \begin{array}{cc}0& 0\\ 1& 1\end{array}& \begin{array}{cc}-2& 0\\ 1& -1\end{array}\end{array}\right].$$

The precision of the phase retardance angle measurement has been estimated from the benchmark measurements for well-known samples and empty polarimeter and established to be better than 1 deg.

## Measurement

The intensity matrix ***I*** has been measured for the 100 J/10 Hz laser chain. The CW alignment beam produced by the Fabri-Parrot laser diode at 1030 nm with polarization maintaining fiber output was expanded and transformed to the flat-top square beam with the dimension of 75 × 75 mm^2^. This CW alignment beam was subject to the PSG before its expansion and injection into the 100 J amplifier. Then it was propagated throughout all four passes of the 100 J amplifier. At the output of 100 J amplifier, the CW beam was reduced by a de-magnification telescope and analyzed by the PSA.

The Mueller matrix was evaluated by the above-described method at the pump rate of 75% of the usual pump power to better account for the lack of energy extraction from the amplifier as the optical-to-optical efficiency of the amplifier is around 25%. The pumping conditions were 3 kW of average power in 1 ms long optical pulses at repetition rate of 10 Hz.

**Figure 3:**
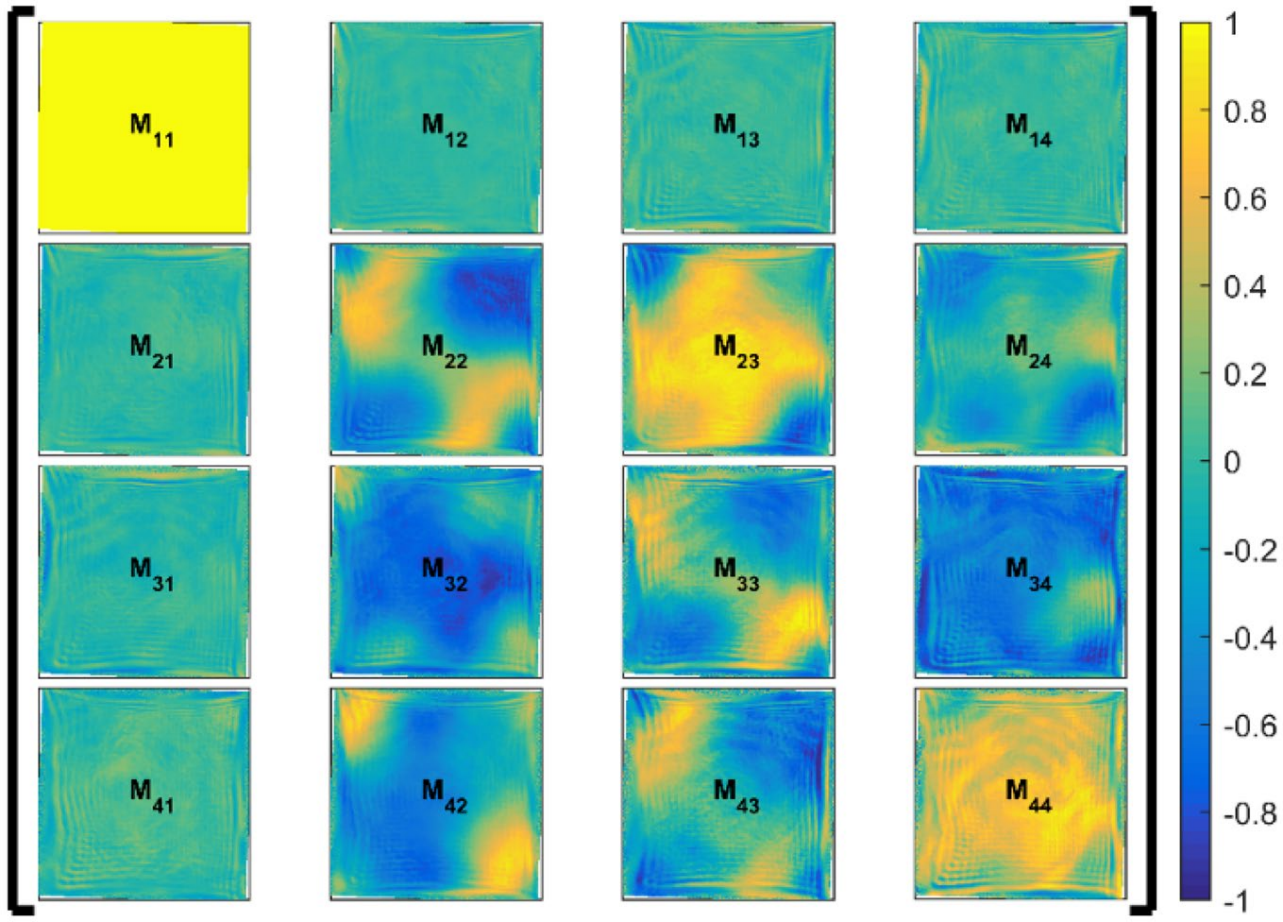
Mueller matrix of the laser system.

The Mueller matrix of the system can be reconstructed from the intensity matrix measurement using (7). The resulting matrix is shown in the Fig. 3.

The Mueller matrix has been normalized so that *M*_*11*_ term is equal to unity over the whole beam cross-section. The normalization has been used in order to suppress the influence of the beam intensity profile. The basic analysis based on the Lu–Chipman polar decomposition ^19^ of the Mueller matrix provides two clear conclusions from the acquired matrix: (1) it can seen directly from the first row of the matrix *M*_*12*_–*M*_*14*_ (containing only the values under the measurement resolution), that no measurable diattenuation is present in the studied part of the laser system; (2) similar conclusion can be drawn from the first column of the matrix *M*_*21*_–*M*_*41*_, which contains the elements proportional to both diattenuation and depolarization. Taking advantage from the knowledge of the diattenuation one can read out also the depolarization present in the amplifier chain. Since the first column signal is under the detection threshold, the laser system shows no depolarization, i.e. the decrease of the degree of polarization towards unpolarized state.

**Figure 4:**
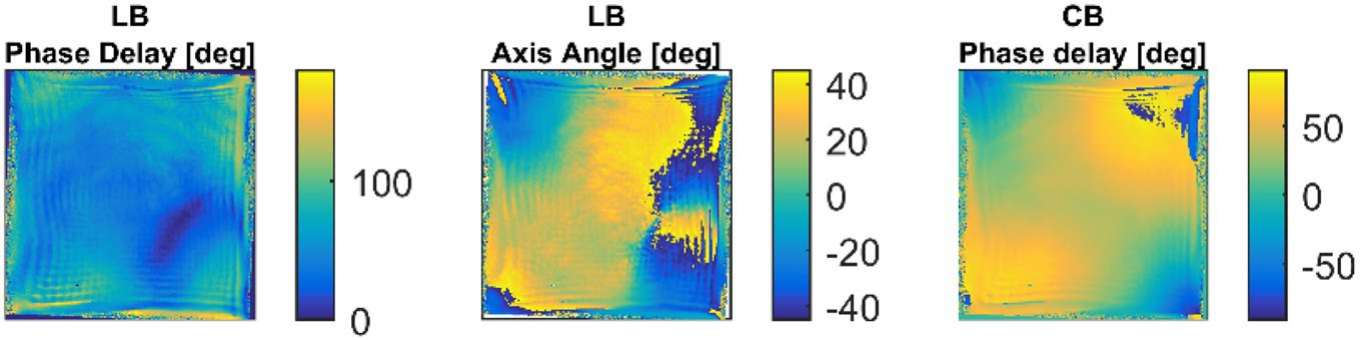
Thermally induced birefringence measured in the laser system which includes also several beam rotations, image-relays and other non-polarization effects. *LB Phase Delay* the phase delay of the linear birefringence, *LB Axis Angle* azimuth of the birefringence axis, *CB Phase Delay* circular birefringence-driven polarization rotation angle.

The information about the birefringence is encoded in the 3 × 3 submatrix located in the right lower corner of the Mueller matrix shown in the Fig. 3. Even if these elements contain also the information about the depolarization and diattenuation, the birefringence can be extracted from them using Lu-Chipman decomposition process. The birefringence obtained from the measured Mueller matrix is shown in the Fig. 4.

It should be noted that according to the original work of Lu and Chipman on polar decomposition, six equivalent realizations of one Mueller matrix exist and are given by different ordering of the depolarizing matrix, diattenuator and retarder matrix, respectively. In our case, however, the depolarizer and diattenuator matrix are very close to the unit matrices and therefore the retarder matrix decomposition is unique within the measurement precision.

## Discussion

**Figure 5:**
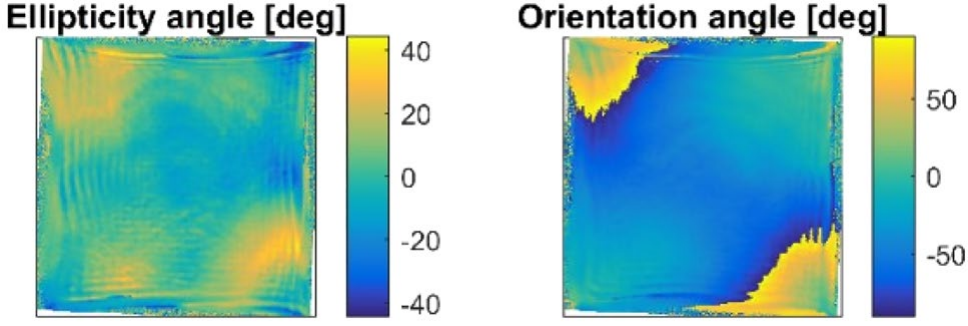
The parameters (ellipticity angle and the inclination angle of the principle axes of the polarization ellipse) of fully pre-compensated laser beam which would produce an unperturbed uniform vertical linear polarization after the propagation through the thermally loaded laser system characterized by the Mueller matrix ***M*** (Fig. 3).

The measured Mueller matrix ***M*** shown in the Fig. 3 can be used for the calculation of the laser system output polarization ***S***_*out*_ for any input polarization state of light ***S***_*in*_ as follows

9 $$S_{out}\left(x,y\right)={\varvec{M}}\left(x,y\right)S_{in}\left(x,y\right),$$

where ***M*** is the non-uniform Mueller matrix obtained from the measurement, ***S***_***in***_ stands for the Stokes vector of the input polarization state, also spatially non-uniform in this case, and ***S***_***out***_ represents the Stokes vector of the laser beam at the end of the amplifier chain. ***S***_***out***_ is desired to be uniformly linearly polarized over the cross-section of the beam. The Eq. (9) was used to search for an ideal input polarization state ***S***_***in***_, which would exactly pre-compensate the thermally induced polarization changes within the laser system represented by ***M***. Such polarization state is shown in the Fig. 5. To the best of our knowledge, such non-uniform polarization state is impossible to generate in current experimental setup as it would have to be created at the main amplifier input, meaning at the output of preceding amplifier, where the typical energy carried by the ns laser pulse is of the order of tens of Joules.

Since the pre-compensated polarization state cannot be created for the final power amplifiers suffering from the most serious thermally induced polarization state changes, one can search for another polarization state, uniform over the cross-section and therefore easy to generate, which would minimize the power loss on the polarizer caused by the thermally induced polarization state changes. It should be noted, that the generalized TIPCL defined in the Fig. 1b is taken into consideration for such input polarization optimization process. The measurement would be then realized using the experimental setup shown in the Fig. 2.There are two ways, how to define TIPCL. The first one is to define it as a ratio of the minimal leakage through the output polarizer to the input power. The second possible definition is convenient for a more complex optical systems which exhibit some non-thermal mechanisms of losses or gain, especially polarization insensitive ones. In such a case it is better to define the TIPCL as a ratio between the minimal leakage through the output polarizer to the power incident on the output polarizer (losses on polarizer are neglected). It should be noted that these two approaches are equivalent, for the optical systems possessing no diattenuation, up to an isotropic multiplicative factor. This factor is not needed for finding the optimum settings and the absolute value of TICPL can be then calculated separately for the previously acquired optimal settings. In our case, the polarization state which minimizes losses has been found by the numerical four-parameter optimization process using the measured Mueller matrix ***M*** shown in the Fig. 3. The optimization process can be formulated as a search for the minimum of the functional *D,* which characterizes the total power loss on the polarizer placed at the end of the amplifier chain extended by the four compensation waveplates according to the Fig. 2. The functional *D* therefore depends on the azimuth vector corresponding to the four waveplates orientation ${\varvec{\phi}}=\left\{{\phi }_{1},{\phi }_{2},{\phi }_{3},{\phi }_{4}\right\}$. The total power loss can be calculated according to the above discussed definition as a ratio of output power ${P}_{out}=\int {I}_{out}\mathrm{d}S$ to total power ${P}_{tot}=\int {I}_{0}\mathrm{d}S$, where the integration is done over the cross-section of the beam. With the usage of (1) and the fact that *A* and *G* vectors do not depend on the spatial coordinates ${P}_{out}\left({\varvec{\phi}}\right)={{I}_{0}A}^{T}\left({\phi }_{3},{\phi }_{4}\right)\int M\left(x,y\right)\mathrm{d}SG\left({\phi }_{1},{\phi }_{2}\right)$. The beam profile intensity *I*_*0*_ is not the function of the spatial coordinates thank to the normalization of the *M* matrix. The direct substitution to the functional *D* gives $D\left({\varvec{\phi}}\right)={A}^{T}\left({\phi }_{3},{\phi }_{4}\right)\langle M\left(x,y\right)\rangle G\left({\phi }_{1},{\phi }_{2}\right)$, where $\langle M\left(x,y\right)\rangle =\int M\left(x,y\right)\mathrm{d}S/\int \mathrm{d}S$. The optimal azimuth vector ${{\varvec{\phi}}}_{opt}$ must satisfy the equation $D\left({{\varvec{\phi}}}_{opt}\right)=\mathrm{min}\left\{D\left({\varvec{\phi}}\right)\right\}$, ${\phi }_{j}\in \langle -\frac{\pi }{4},\frac{\pi }{4}\rangle , \forall j=1,.., 4$. In our case, the optimal ${\varvec{\phi}}$ vector has been found to be ${{\varvec{\phi}}}_{opt}=\left\{-0.166, 0.579, 0.658, -0.674\right\}$ rad.

**Figure 6:**
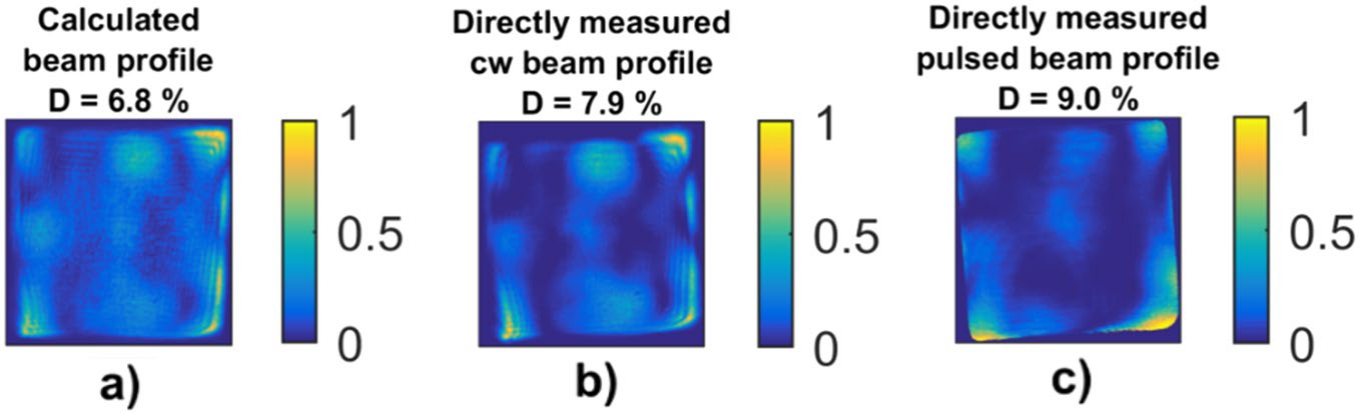
The polarizer-rejected part of the beam with polarization state non-uniformly changed by thermal-stress-induced birefringence. The beam profiles shown are (**a**) calculated using Mueller matrix Fig. 3, (**b**) measured using CW alignment beam, (**c**) measured using the pulsed beam with no amplification.

The resulting input polarization state which should be generated at the amplifier chain input is the left-handed elliptical polarization with the ellipticity angle of ${\chi }_{opt}=37.7 \mathrm{deg}$ and the azimuth of ${\theta }_{opt}=33.2 \mathrm{deg}.$ The total power loss caused by the thermal-stress-induced polarization change has been reduced from more than 33% to less than 10%. The initial power loss was measured under the same pumping conditions when input polarization was set to linear horizontal and ratio of output horizontal and vertical polarization was measured. It can be noted that the input polarization state optimization, while leaving the output polarizer unchanged resulted in the power loss decrease to 26% only. The predicted compensation has been tested experimentally with ‘Bivoj’ laser system. Two waveplates were used at the amplifier chain input to generate the desired polarization state with ${\chi }_{opt}$ and ${\theta }_{opt}$. Another two waveplates were placed at the amplifier output to transform the polarization to linear again. The comparison of the computed and directly measured polarizer-rejected part of the beam is shown in the Fig. 6.

To confirm that the system will behave in the same way when we will be amplifying the pulsed beam, pulses from 10 J amplifier with energy of 7 J at repetition rate of 10 Hz were injected into the main power amplifier. These pulses were amplified in 100 J final amplifier to 85 J. Pump power used was 4 kW in 1 ms long pulses at repetition rate of 10 Hz. The waveplates orientations obtained from CW experiment were maintained and only the depolarization loss measurement was performed. The depolarization loss increased to 9%. The polarizer-rejected beam profile is shown in the Fig. 6 c). The power loss due to the thermal-stress-induced birefringence with the pulsed beam slightly increased, from 6.8 to 9%, compared to the values calculated from the Mueller matrix of the amplifier chain. We attribute this difference to the fact that the Mueller matrices have been measured using the CW alignment beam and not the pulsed beam. The difference occurred especially at the edges of the beam, where there was some diffraction visible in the CW beam profile, while this was not present in the pulsed beam.

The thermal phenomena do not change significantly during the laser operation, because they are related to the overall stability of the laser, for which the system is designed. From our own experience, there is no need for any tuning of the compensation during the laser run. The TIPCL changes during the laser run do not exceed 1%, however, taking into account the measurement precision of the in-situ power monitoring, this fluctuation lies within the accuracy of the measurement. Some fine tuning of the output waveplates Q_2_, H_2_ (less than 1 deg) is needed after the laser operation pause e.g. during the night break. However, another polarimetric analysis was not needed so far and the compensation quality is maintained exclusively by these fine tunings. Our experience with the compensation stability is based on the second harmonic generation experiments which are very sensitive to the polarization changes within the beam profile.

## Data Availability

The datasets analyzed during the current study available from the corresponding author on reasonable request.
